# Evaluation of Comorbidities in Patients with OSAS and Simple Snoring

**DOI:** 10.1155/2013/709292

**Published:** 2013-04-18

**Authors:** Ömer Hızlı, Müge Özcan, Adnan Ünal

**Affiliations:** ^1^Giresun A. İlhan Özdemir State Hospital, 28100 Giresun, Turkey; ^2^Ankara Numune Education and Research Hospital, Ankara, Turkey

## Abstract

*Aim*. OSAS is a disease characterized by repetitive air flow constraint or cessation due to airway collapse. Diseases that frequently coexist with OSAS and simple snoring were evaluated in this study. *Materials and Methods*. This study was conducted in the Otorhinolaryngology Department of the Ankara Numune Hospital between April 2008 and April 2010 with 130 patients who presented with the complaints of snoring, witnessed apnea, and daytime drowsiness. Presence of chronic disease was compared to the demographics, BMIs, Epworth Scale scores, polysomnography, and physical examination findings. *Results*. Comorbid diseases were present in 56 (43.1%) of the patients, and the most presented disease group was cardiovascular system diseases. Age, BMI, daytime drowsiness, and frequency of septum deviation were observed at higher rates in patients with chronic disease. Age, BMI, and frequency of septum deviation were associated with cardiovascular diseases. Endocrine disease was found to increase with decreased oxygen saturation. Neuropsychiatric diseases were associated with daytime drowsiness and age. The mean age was lower in cases with cigarette smoking compared to cases without. *Conclusion*. Frequency of the comorbidities mostly increased with age as expected. Comorbid diseases were also associated with obesity and daytime drowsiness. Cigarette smoking was associated with early-age disease.

## 1. Introduction

Obstructive sleep apnea syndrome (OSAS) is a disease characterized by repetitive air flow constraint or cessation due to airway collapse, which may be evident as simple snoring, while patients might present with a series of symptoms that occur in serious cardiac and pulmonary complications [1–5]. OSAS has been known to be associated with congenital diseases, upper airway pathologies, cardiovascular diseases, lung diseases, gastrointestinal system diseases, endocrine diseases, collagen tissue diseases, and neuropsychiatric diseases [6]. The conditions associated with OSAS, such as hypertension, diabetes mellitus, coronary artery disease, myocardial infarct, and congestive cardiac failure, are etiological risk factors for OSAS at the same time [7].

We evaluated the associations of OSAS and comorbid diseases in patients diagnosed with OSAS and simple snoring presenting to the Sleep Disorders Center of the Ankara Numune Education and Research Hospital. Demographics of the patients, body mass index (BMI), Epworth Sleepiness Scale (ESS) scores, polysomnography (PSG) parameters, physical examination findings, and patients' responses to some questions specifically prepared for this group of patients, as well as the associations of OSAS and cardiovascular, pulmonary, endocrine, gastrointestinal, and neuropsychiatric diseases, and cigarette smoking were analyzed. 

## 2. Materials and Methods

This study was conducted in the Otorhinolaringology Department of the Ankara Numune Education and Research Hospital between April 2008 and April 2010 with 130 patients who presented to the hospital with the complaints of snoring, witnessed apnea, and daytime drowsiness. Responses to three questions in particular during the first examination were included in the evaluation. Do the people around you tell that you snore? Do the people around you tell that your breathing stops? Are you in need of excess sleep during the day? 

During the first examination, responses were recorded to questions regarding the presence of cigarette smoking, asthma, other lung diseases such as chronic obstructive lung disease, hypertension, diabetes, endocrine diseases, such as goiter and hypothyroidism, cardiologic diseases, such as hypertension, hyperlipidemia, atherosclerotic cardiac disease, and congestive cardiac failure, neuropsychiatric diseases, such as depression, anxiety, Parkinson's disease, epilepsy, and insomnia, and gastroesophageal reflux disease. 

 The ESS, which was translated from the original text, was used to evaluate excess sleepiness status [8].

The height and weight of the patients were measured and BMI of the patients was calculated as body weight (kg)/height (m^2^). 

Detailed ear, nose, and throat examinations and endoscopic examinations were performed in all patients, and pathologies of the nose, oral cavity, nasopharynx, hypopharynx, and larynx, if any, were recorded. 

PSG was applied to all patients in a single-bedded room, with a supervising technician during spontaneous sleep in the Center for Snoring and Sleep Disorders of the Ankara Numune Education and Research Hospital. During the PSG, an electroencephalogram (EEG), electromyogram (EMG-submental), electromyogram (EMG-right/left tibialis), electrooculogram (right-left EOG), electrocardiography (ECG), nasal air flow, thoracic and abdominal respiratory movements, blood oxygen saturation with pulse oximetry, and body position parameters were recorded throughout the night. 

The distribution of stages of sleep according to the scoring, duration of total sleep, rapid eye movements (REM) duration, non-REM duration, the number and maximum duration of respiratory events during this period, apnea-hipopnea index (AHI) of the entire night, AHI at REM and non-REM, lowest oxygen saturation, arousal index, AHIs lying on the right and left sides, and in the prone and supine positions were recorded. 

Demographics of the patients, PSG findings, and physical examination findings were determined. The relation between chronic diseases and demographics, PSG findings and physical examination findings were analyzed by the univariate logistic regression analysis, Pearson's chi-square, or Fisher's exact chi-square tests. The multivariate logistic regression analysis technique was used to determine the distinguishing factors used to compare the group with chronic disease versus the group without.

## 3. Results

Ninety of the patients were males (69%) and 40 (31%) were females with an age range of 27–95 years (mean: 47 years). BMI results of patients were between 20.6 and 51.2 and mean was 29.2. According to the PSG results AHI levels of patients were between 0.2 and 124.3, and the mean was 22.4. Simple snoring was determined in 26 (20%) of patients, mild OSAS was determined in 40 (30.77%) of patients, moderate OSAS was determined in 33 (25.38 %) of patients, and severe OSAS was determined in 31 (23.85%) of patients.

Comorbid diseases were present in 56 (43.1%) of the patients; 10 (7%) had lung diseases, 13 (10%) had neuropsychiatric diseases, 17 (13.1%) had endocrine diseases, 28 (21.5%) had gastroesophageal reflux disease, and 29 (22.3%) had cardiovascular system diseases.

Presence of chronic diseases was compared to other parameters statistically. As a result of these comparisons, mean age, BMI, and daytime drowsiness with a chronic disease were higher compared to the cases without chronic diseases. In addition, the prevalence of septum deviation was higher in patients with a chronic disease compared to those without a chronic disease. 

The mean age, mean BMI, and prevalence of septum deviation were higher in cases with cardiologic disease compared to the cases without a cardiologic disease. Septum deviation was seen more frequently in patients with gastroesophageal reflux compared to the cases without reflux. Mean age was higher in cases with an endocrine disease compared to the ones without an endocrine disease. Mean age was statistically significantly high in cases with a neuropsychiatric disease compared to the cases without a neuropsychiatric disease. 

Daytime drowsiness was higher in the group with neuropsychiatric diseases compared to the group without diseases. 

The mean age was lower in cases with cigarette smoking compared to those who did not smoke. 

## 4. Discussion

OSAS has been reported to be seen between the ages of 40–65 years; gender and obesity are the most prominent risk factors [9, 10]. It is reported that its prevalence demonstrates an increase during the ages of 18–45 years and a plateau between 55–65 years; AHI levels also increase with age [11, 12]. Mean age of the patients was found to be 47.7 ± 9.9 years in our study. In addition, the incidence of the presence of a chronic disease was found to increase with age as expected.

In a study performed by Gabbay and Lavie with 23,806 patients in 2012, frequency in males and females of lung diseases, cardiovascular diseases, hypertension, diabetes mellitus, and dyslipidemia was 9% and 12.4%, 13.2% and 7.1%, 40.1% and 43.6%, 3%, and 2.4%, and 23.6% and 25.5%, respectively [13]. In our study, comorbid diseases were present in 56 (43.1%) of the patients; 10 (7%) had lung diseases, 13 (10%) had neuropsychiatric diseases, 17 (13.1%) had endocrine diseases, 28 (21.5%) had gastroesophageal reflux disease, and 29 (22.3%) had cardiovascular system diseases. 

Ursavaş et al., in a study performed in 2004, found that 44 (36.9%) out of 119 patients (105 males and 14 females) who were diagnosed as sleep apnea syndrome by PSG were overweight (BMI: 26–39) and 48 (40.5%) were obese (BMI > 30) [14]. Although BMI is higher in men compared to women, the severity of OSAS is less in women compared to men with the same BMI, which suggests a gender-related difference. This can be attributed to the different fat distribution in the two sexes [15]. The mean BMI was 29.8 ± 4.5 in our study with a mean BMI of 29.0 ± 4.1 and 30.8 ± 4.9 in cases without and with a chronic disease. The increase in the BMI was shown to increase the incidence of chronic diseases in patients with OSAS and simple snoring as expected. 

ESS scores and the presence of chronic diseases were not found to be associated, and this was found to be correlated with the literature findings [16]. The association between OSAS and cardiovascular diseases has long been known and has been demonstrated clearly in some previous case-control studies [17]. Our study is planned not to demonstrate the increased cardiovascular disease risk in patients with OSAS but to identify the causes of this fact. Cardiovascular diseases seen in 22.3% (29 cases) of the patients were found to be affected by age, and the incidence of cardiovascular disease increased with the increasing age of the patients with OSAS and simple snoring. Since cardiovascular disease risk is also increased in the normal population, it is unknown if this situation is significant in patients with OSAS and simple snoring. In addition, the incidence of septum deviation in patients was found to increase in cases with cardiologic diseases. Septum deviation was also observed to be associated with gastroesophageal reflux but this relationship is not clear. 

Impaired glucose tolerance, metabolic disorders, and dyslipidemia may be seen in patients with OSAS [18]. The incidence of those endocrine diseases was found to increase with age in our study. Since diabetes mellitus risk is also increased in the normal population, it is unknown if this situation is significant in patients with OSAS and simple snoring. In a review by Aloia et al. in 2004, sleep disturbances and hypoxemia were reported to have an effect on mood; however, they suggested that daytime drowsiness instead of hypoxemia occurring at night predicted the severity of depression and anxiety seen in patients with OSAS [19]. In this present study, 10% (13 patients) of the cases had neuropsychiatric diseases and the incidence of neuropsychiatric diseases was higher in patients with daytime drowsiness.

Although cigarette smoking is known to increase the upper airway resistance by producing inflammation, edema and mucus secretion, the association between cigarette smoking and OSAS is not clear [15]. The lower mean age of the patients with cigarette smoking suggests that cigarette smoking results in an earlier onset age of OSAS, thus contributing indirectly to the morbidity of OSAS. 

## 5. Conclusion

Frequency of the comorbidities seen in patients with OSAS and simple snoring mostly increased with age as expected. BMI, daytime drowsiness, and septum deviation were found to be higher in cases with chronic diseases compared to cases without a chronic disease. The association of BMI and presence of chronic disease suggests that weight loss may decrease comorbidities in patients with OSAS and simple snoring. The incidence of cardiovascular, endocrine, and neuropsychiatric diseases increases with advanced age as expected. Obesity and cardiovascular disease were found to be associated in patients with OSAS. On the other hand, neuropsychiatric diseases were associated with daytime drowsiness. The coexistence of daytime drowsiness with neuropsychiatric diseases suggests that neurological and psychosocial improvement might be achieved in these patients with the treatment of OSAS. According to our findings the low mean age of cases with cigarette smoking suggests that cigarette smoking lowers the age of peak incidence of OSAS and thus it was suggested to contribute to the OSAS-associated comorbidities.
